# Physicians’ knowledge, attitude and practice of clinical audit in a tertiary health facility in a developing country: a cross-sectional study

**DOI:** 10.11604/pamj.2022.43.22.33800

**Published:** 2022-09-14

**Authors:** Ezioma Anne Alinnor, Daprim Samuel Ogaji

**Affiliations:** 1 School of Public Health, University of Port Harcourt, Port Harcourt, Nigeria; 2 Africa Centre of Excellence in Public Health and Toxicological Research, University of Port Harcourt, Port Harcourt, Nigeria

**Keywords:** Physicians, knowledge, attitude, practice, clinical audit, University of Port Harcourt Teaching Hospital

## Abstract

**Introduction:**

health outcomes in hospitals can be improved through regular conduct of clinical audit. This study assessed physicians´ knowledge, attitude, and practice of clinical audit at the University of Port Harcourt Teaching Hospital (UPTH) in Rivers State.

**Methods:**

a descriptive cross-sectional study involving 460 doctors selected through convenience sampling. A pretested self-administered questionnaire was administered, and data was analysed using the Statistical Package for Social Research (SPSS) 23.0.

**Results:**

a total of 457 questionnaires were analyzed giving a response rate of 99.3%. Only 57 (12.5%) of the 457 respondents had a correct understanding of the clinical audit process. Most respondents (75.1%) agreed that clinical audit is important in improving patient care, however only 29.9% were aware that the hospital has a clinical governance structure. Seventy-three (16.0%) doctors had received training in different forms of clinical audit, while 148 (33.0%) were involved in different clinical audit activities, with mortality audit being the most common clinical audit type (81, 17.7%).

**Conclusion:**

physicians at the University of Port Harcourt Teaching Hospital have poor understanding of the clinical audit process. The integration and scale-up of clinical audit activities as part of an overall clinical governance system in the teaching hospital is recommended.

## Introduction

Clinical audit (CA) is defined as “*a systematic critical review of the quality of medical care, encompassing diagnostic and treatment procedures, resource utilization, and the patient´s outcome and quality of life*” [1]. CA is now an important aspect of clinical professional practice, since it entails comparing current practice to evidence-based best practice in the form of standards of care, identifying areas for quality improvement, and making changes to meet these standards [2]. Undertaking CA requires a collaborative and methodical framework for improving patient care, allows for an objective examination of healthcare delivery processes and guarantees that healthcare workers assess their practice in a systematic fashion by identifying and encouraging appropriate practices while highlighting inefficient procedures, resulting in better patient-care systems [2].

The focus of service delivery has transitioned from disease cure and treatment to a more holistic approach that includes different areas of care. With the rapid expansion of healthcare, it is very essential to monitor, examine, and evaluate the various health services available [3]. It is also critical for health care providers to participate in routine and systematic medical and clinical audits, to properly record data, and to use the audit results to enhance their practice. This is because it serves as a reference and overview of the quality of care supplied to patients in comparison to set standards. Any deviation from best practices can then be investigated to determine the root cause and make changes to remedy the situation [2].

Doctors, as heads of the health team, play significant roles in the delivery of health care services and must be held accountable for their clinical activities. In Nigeria, medical litigation cases are on the increase due to factors ranging from increased incidences of medical malpractice/negligence among poorly trained doctors, increasing awareness of patients´ rights and even increased interest by lawyers in such cases [4]. This is in-spite of the codes of medical ethics and the laws that regulate and guide the practice of medicine [5]. It is therefore pertinent to incorporate a systematic CA program into health care delivery given the numerous benefits that this process brings at all levels [1-3].

The efficient implementation of CA in a health institution advances the provision of excellent health care in an ever-changing health-care environment and ensures that patients are satisfied with the health services provided in the health institution [6,7]. Clinical auditing ensures accountability and demonstrates health professionals´ attempts at providing high-quality care to patients. As a tool for positive change, it will lead to reduced medical litigation since standards of care can be monitored and followed up at regular intervals [8]. To effectively apply the clinical audit process in clinical practice, doctors must first understand what it entails and how the overall goal of service improvement can be met and they must be receptive to the use of CA to improve the quality of health care given [9,10]. A proper CA process requires a systematic evaluation of the components of the organization, practices, and results of care against specified standards [11,12]. In a clinical context, the audit process aids in the detection of inadequacies that may be addressed in order to provide evidence-based best clinical practice and high-quality health care [13].

Three broad components captured during CA include measuring specific elements of clinical practice (measurement), comparing results with recognized standards (comparison) and reflecting on the outcome of audit with the intent of incorporating changes to practice accordingly (evaluation) [12]. CA are of various types such as standard-based audits, adverse occurrence screening and clinical events monitoring audits, peer-review audits and focus-group and patient surveys among others. A clearly stated audit objective provides the audit project team with a specification of the audit's purpose and scope, mutual knowledge of the audit´s goals, and proper notification of the audit´s scope and purpose to others [13].

CA as a specialized form of quality assessment may involve the structure-process-outcome triad of the system for delivering health care [14]. Classically, CA adopts a cyclical stepwise process involving different steps which are: identifying the need for CA (should be of high priority such as complaints or adverse events), the definition of criteria/standards (using recommendations from clinical practice guidelines and must reflect current practice); a collection of data on the structure, process and/or outcomes and analysis of data (tightly directed by established objectives and in line with ethical considerations); comparing performance with standards (using pre-defined criteria to ensure process quality control); recording gaps and inefficiencies, suggesting/providing recommendations and implementing change (stating clear action plans for effective implementation ) and finally re-auditing (for follow-up comparison after implementing suggested changes) [11-14]. Physicians´ knowledge, attitude and practice (KAP) surveys are aimed at collecting information on what is known, believed and done by doctors as it relates to a specific topic. Data obtained about physicians´ knowledge is used to assess the level of information they have about basic concepts [15]. Attitude is defined as a learned disposition to think, feel and act in a particular way or towards a particular object/issue at hand [16]; and gives an overview of acceptability or not of a given concept; while questions pertaining to practice enquire about behaviour and thus yields information about what is done or what should be done [17]. KAP surveys are necessary because, in health care practice, ignorance is not the sole reason for deficiencies in health care, but rather, failure to apply what has been learnt.

Audit is an integral part of clinical practice in developed countries but it is yet to attain a similar position in developing countries. The need for a structured programme of CA is thus long overdue [18]. Cheater and Keane[19] reported that knowledge of clinical audits was sparse among healthcare professionals, with the attendant consequence of limiting the extent to which they make use of clinical audits in healthcare service delivery. Following a post-course evaluation among Family Medicine trainees in Kuwait, the majority were shown to understand the essential steps for carrying out an audit, as a result of being exposed to regular audit projects [20]. A survey in the United Kingdom showed a largely positive attitude of veterinary surgeons toward CA as most respondents agreed that it helped them enhance their clinical standards and advance their careers [21]. Kediegile and Madzimbamuto [22] reported that negative attitudes towards CA included worries about victimization following audit findings and concerns about having insufficient resources to apply the adjustments made. Perceived disadvantages of CA according to a review of 93 publications included diminished clinical ownership, restriction of clinical freedom, and fear of litigation [23]. A study in a tertiary care centre in Nigeria showed statistically significant improvements in the standard of care of four clinical conditions as well as clinical monitoring and drug use following a CA of obstetric care received by patients [24].

This baseline study was designed to assess the knowledge, attitude, and practice of clinical audit among physicians at the University of Port Harcourt Teaching Hospital (UPTH) in Rivers State.

## Methods

**Design of the study:** this was a descriptive cross-sectional study.

**Study area:** the study was carried out at the University of Port Harcourt Teaching Hospital (UPTH), in Obio-Akpor Local Government Area (LGA) of Rivers State, Nigeria. UPTH is a research facility and training centre for health workers at the undergraduate and postgraduate levels. It provides essential health services to patients within the state and serves as a referral centre for neighbouring states including Bayelsa, Abia, Akwa-Ibom and Imo. It is run by a three-tiered management structure that includes the Board of Management, the Hospital Management Committee, and the different departments. It is an 800-bed multi-specialist hospital with clinical specialities having wards for in-patient management, ambulatory and emergency care. Paediatrics, Internal Medicine, Surgery, Dentistry, and Obstetrics and Gynaecology are the main clinical departments. Other clinical departments are Ophthalmology, Family Medicine, Ear, Nose and Throat (ENT), Radiology, Neuropsychiatry, Anaesthesia and Pathology. Doctors, nurses, radiographers, dieticians, physiotherapists and laboratory scientists are among the health human resources employed at the University of Port Harcourt Teaching Hospital [25].

**Study population:** as of the year 2020, the University of Port Harcourt Teaching Hospital had 695 doctors in various specialities comprising 200 consultants, 460 residents and house officers. Their ages range from 20-70 years and they comprise both male and female doctors.

**Study procedure:** prior to the commencement of the study, advocacy visits were made to the Heads of departments/Chief Residents to intimate them about the study, seek their cooperation, obtain permission, and notify clinical staff in the departments. Web-based and direct administration of questionnaires were deployed. For the direct administration, specific days coinciding with departmental activities e.g., mortality meetings, seminar presentations etc were identified weekly to administer the questionnaire to the doctors in each department. Each doctor who agreed to participate in the study was given a self-administered questionnaire and it was collected afterwards. Any doctor who was unavailable on the appointed day was communicated with, and a convenient time was agreed on for the purpose of administering the questionnaire. Questionnaire administration was conducted between 30^th^ April and 30^th^ May 2021.

### Eligibility criteria

***Inclusion criteria:*** doctors who work in UPTH clinical departments.

***Exclusion criteria:*** doctors who were unable to participate in the study or who declined participation. Incomplete questionnaires with up to 30 percent of unanswered questions.

**Sampling method:** convenience sampling method was employed in this study with the goal of reaching as many doctors as are available at their duty posts during the survey. The different specialities offering health care services at UPTH were - Ear Nose and Throat, Radiology, Surgery, Dentistry, Ophthalmology, Anaesthesia, Paediatrics, Obstetrics and Gynaecology, Family Medicine, Pathology, Mental Health, Community Medicine and Internal Medicine departments. The list of doctors in the different working cadres (consultants, senior registrars, registrars and house officers) in each of the specialities was then obtained. This was to enable a fair representation during the administration of the questionnaires.

**Data source/study instrument:** a self-administered semi-structured questionnaire was used as the research tool. There were four sections to the study questionnaire which described the variables to be studied as follows: Section A captured the socio-demographic data of the respondents including their gender, age, department, years of practice and cadre. Section B probed their knowledge of clinical audit. Section C elicited data on the attitude of the respondents towards clinical audit. Section D probed the practice of clinical audit among the doctors.

**Validity/ reliability of study instrument:** prior to commencement of data collection, the study instrument was pretested among 30 doctors at the Rivers State University Teaching Hospital (a tertiary centre in a different LGA- Port Harcourt LGA, in Rivers State) to ascertain the feasibility/appropriateness of the methodology and improve on likely areas of limitations. Required changes were made following the pretest and the internal consistency reliability measure using the Cronbach´s alpha coefficient was 0.853.

**Determination of sample size:** with the dearth of previous local studies on the subject, a proportion of 50% of doctors with adequate knowledge of CA was assumed. The minimum sample size of 384 participants in this study was calculated using the formula


$$n=\frac{Z_{\alpha^{2}}pq}{d^{2}}$$

where: Z_Þ_(standard normal deviation corresponding to selected level of 0.025 in each tail) = 1.96; n = sample size, p = proportion of physicians with adequate knowledge of CA = 50% (0.5); q= 1 - p = 1 - 0.5 = 0.5, e = precision of 5% at 95% degree of confidence. A 20% upward adjustment for the calculated sample size was done to provide for non-response or inappropriately entered data bringing the total sample size to 460 respondents.

**Data analysis:** the Statistical Package for Social Sciences (SPSS) version 23.0 software was used to analyze the data. Absolute and relative frequencies were computed and presented in tables. For assessment of knowledge of the CA process, participants were expected to arrange the various CA steps in sequence. Only those who correctly identified all steps in the sequence of CA were regarded as having correct knowledge of the CA process. Assessment of attitude and practice was obtained from their responses to questions asked in the appropriate sections of the study instrument.

**Ethics, consent, and human right:** ethical clearance (UPH/CEREMAD/REC/MM74/103) was obtained from the Research and Ethics Committee of the University of Port Harcourt Teaching Hospital (UPTH) before the commencement of the study. Informed consent was obtained from all doctors recruited for the study. Participation was voluntary and participants were allowed to withdraw their participation at any point if they so desired. Anonymity and confidentiality were upheld throughout the study.

**Funding:** this study was funded by the authors.

## Results

A total of 460 questionnaires were returned with 457 of them correctly filled, yielding a response rate of 99.3%. Three questionnaires were incompletely filled and were not analyzed as they had more than thirty percent of questions unanswered.

From Table 1, the male-to-female ratio of physicians in this study was 1: 1. Majority were below 40 years of age (n = 314, 68.7%) and had practised for between 1-9 years (227, 49.7%). Registrars made up the largest group of respondents (149, 32.5%) and the highest rate of responses were from the departments of Medicine (30.6%) and Surgery (31.1%). Table 2 presents data on the assessment of the physicians´ knowledge of clinical audit. The majority were aware of clinical audit (295, 66.7%). However, only 57 (12.5%) of them correctly identified the sequence of activities in the clinical audit cycle or were aware of the structure for clinical audit in the hospital (29.9%).

**Table 1:** sociodemographic characteristics of respondents

Variable	Category	Frequency (%)
Sex	Female	230 (50.3)
	Male	227 (49.7)
Age	<40years	314 (68.7)
	40-60years	134 (29.3)
	>60years	9 (2.0)
Department	Medicine	140 (30.6)
	Surgery	142 (31.1)
	Obstetrics & Gynaecology	46 (10.1)
	Paediatrics	71 (15.5)
	Laboratory Medicine	58 (12.7)
Years in Practice	1-9	227 (49.7)
	10-19	180 (39.4)
	≥20	50 (10.9)
Cadre	Interns	77 (16.8)
	Registrars	149 (32.6)
	Senior. Registrar	129 (28.2)
	Consultant	102 (22.3)

**Table 2:** knowledge of clinical audit

Variable	Frequency (%)
General knowledge of CA	295 (66.7)
Correct knowledge of the CA process	57 (12.5)
Identifies CA as assessment of frequency and volume of service provision	373 (87.1)
Identifies CA as assessment of risks associated with provision of health care	367 (86.8)
Identifies CA as assessment of effectiveness of interventions during health care delivery	431 (99.1)
Aware of a structure of CA in the hospital	135 (29.9)
Aware of a CA policy in the hospital	94 (20.8)
Aware of a CA team in the Department	102 (22.7)

As shown in Table 3, majority of the respondents strongly agreed that clinical audit leads to improvement in patient care (241, 54.0%), improves patient satisfaction (213, 47.8%), encourages efficient use of resources (233, 52.1%) and improves teamwork (172, 38.8%). The majority also strongly disagreed that clinical audit diminishes clinical ownership (100, 22.8%), instigates hierarchical suspicion (131, 29.8%) and creates professional discord (187, 42.0%). Similarly, more of the respondents strongly inclined to hospitals having clear policies on CA (285, 63.8%), central coordinating structure for CA (228, 51.0%), and clinical departments should have a CA team/committee (253, 56.6%). Majority also strongly agreed that CA team should be multi-disciplinary and include non-medical staff (168, 37.9%), CA be included in undergraduate medical education (158, 35.7%) and form part of annual Continuing Medical Education for doctors (152, 34.4%). Table 4 presented data on the involvement of physicians in CA activities, showing only 73 (16.0%) of physicians had received training in clinical audit, commonly on collecting data (52, 11.4%), identifying audit needs (48, 10.5%), defining methodology (43, 9.4%), analyzing data (42, 9.2%), setting standards (38, 8.3%), creating action plans (37, 8.1%) and implementing change (36, 7.9%). Mortality audit was the most commonest form of CA physicians were involved in (81, 17.7%) and their involvement was when it was required of them (96, 21.0%).

**Table 3:** attitude towards clinical audit

Statements in CA	Response				
	Strongly disagree Freq (%)	Disagree Freq (%)	Neutral Freq (%)	Agree Freq (%)	Strongly agree Freq (%)
CA leads to improvement in patient care	52 (11.4)	16 (3.6)	43 (9.6)	94 (21.1)	241 (54.0)
CA improves patient satisfaction	50 (11.2)	15 (3.4)	33 (7.4)	135(30.3)	213 (47.8)
CA encourages efficient use of resources	42 (9.4)	21 (4.7)	26 (5.8)	125 (28.0)	233 (52.1)
CA improves knowledge of patient care	38 (8.6)	32 (7.2)	53 (12.0)	124 (28.1)	195 (44.1)
CA increases job satisfaction	44 (9.9)	36 (8.1)	97 (21.8)	132 (29.7)	136 (30.6)
CA improves teamwork	39 (8.8)	29 (6.5)	64 (14.4)	139 (31.4)	172 (38.8)
CA diminishes clinical ownership	100 (22.8)	99 (22.6)	127 (29.0)	65 (14.8)	47 (10.7)
CA instigates hierarchical suspicion	131 (29.8)	109 (24.8)	118 (26.9)	54 (12.3)	27 (6.2)
CA restricts clinical freedom	131 (30.0)	138 (31.6)	87 (19.9)	49 (11.2)	32 (7.3)
CA creates professional discord	187 (42.0)	136 (30.6)	50 (11.2)	40 (9.0)	32 (7.2)
CA impedes individualized care	163 (35.7)	131 (28.7)	76 (16.6)	41 (9.0)	35 (7.7)
Hospitals should have clear policy on CA	33 (7.4)	15 (3.4)	30 (6.7)	84 (18.4)	285 (63.8)
Hospitals having central coordinating structure for CA	35 (7.8)	25 (5.6)	42 (9.4)	117 (26.2)	228 (51.0)
Clinical department having clinical audit team/committee	30 (6.7)	18 (4.0)	31 (6.9)	115 (25.7)	253 (56.6)
CA team should include only doctors	190 (42.5)	113 (25.3)	80 (17.5)	20 (4.5)	44 (9.8)
CA should involve non-medical staff	46(10.4)	38 (8.6)	63 (14.2)	128 (28.9)	168 (37.9)
CA should be included in undergraduate medical education	42 (9.5)	39 (8.8)	76 (17.2)	127 (28.7)	158 (35.7)
CA should be part of the annual CME for doctors	37 (8.4)	33 (7.5)	83 (18.8)	137 (31.0)	152 (34.4)
CA should be a routine task of all doctors	45 (10.2)	42 (9.5)	99 (22.4)	136 (30.8)	120 (27.1)

**Table 4:** practice of clinical audit

Variable	Responses	Frequency (%)
Physicians trained in CA		73 (16.0)
Aspects of CA Process trained in:	Identifying audit need	48 (10.5)
	Setting standards	38 (8.3)
	Defining methodology	43(9.4)
	Collecting data	52 (11.4)
	Analyzing data	42 (9.2)
	Creating Action plan	37 (8.1)
	Implementing Change	36 (7.9)
Physician involvement in forms of CA		148 (33.0)
Form of CA involved in:	Mortality audit	81 (17.7)
	Patient satisfaction survey	26 (5.7)
	Process audit	19 (4.2)
	Adverse events monitoring	9 (2.0)
	Treatment outcomes	24 (5.3)
	Cost of care	12 (2.6)
	Reflective practice	18 (3.9)
Frequency of CA activities	Daily	8 (1.8)
	Weekly	36 (7.9)
	Monthly	35 (7.7)
	Annually	11 (2.4)
	When required	96 (21.0)
	Never	265 (58.0)

## Discussion

The study determined the level of knowledge, attitude, practice, and other characteristics related to clinical audits among doctors at the University of Port Harcourt Teaching Hospital. For clinical audit to be used effectively in health care, health professionals, including doctors, must understand what it involves and how it can be accomplished. An assessment of clinical audit knowledge was conducted in this study based on this concept. Although the majority of the respondents had some general knowledge of clinical audit, only a few could accurately state the steps of the clinical audit cycle, which was utilized as an objective assessment of measuring their knowledge. This was in contrast to a cross-sectional study in the United Kingdom that examined the experiences and attitudes of veterinary surgeons toward clinical audit, in which the majority of them were also able to correctly describe the clinical audit process, in addition to having heard about it [21]. Another study in Kuwait found that the majority of Family Medicine trainees had a strong comprehension of clinical audit [20]. The fact that only a small percentage of respondents were aware of the steps in the clinical audit process suggests that it has the potential to limit the extent to which health professionals may employ clinical audit concepts in quality health care service delivery and assessments [19].

In this study, the attitude of the respondents towards various aspects of clinical audit was assessed. The majority of the respondents in this study had a positive attitude about the various areas of clinical audit that were investigated. The majority of the respondents in this survey believed that the clinical audit process should be interdisciplinary and include other members of the health team, which was one of the most important findings. They believed that implementing a clinical audit system would result in enhanced patient care, patient satisfaction, resource efficiency, and teamwork. This finding corroborates with that of the study conducted by Waine *et al*., [21] in the United Kingdom on the attitudes of veterinary surgeons towards clinical audit, which found that their attitudes towards clinical audit were generally positive. This was because the majority of the respondents agreed with statements that clinical audit improved clinical standards in veterinary practice and aided their professional development [21]. Johnston *et al*., [23] in another study also reported that improved communication and job satisfaction were some benefits gained from participating in clinical audit.

Respondents in the former study were however concerned about the presence of available resources necessary to implement changes recommended from clinical audits, the current workload being borne by doctors, technical know-how in conducting clinical audit, presence of clinical audit committees in their department/hospital, availability of time for clinical audit activities as well as the threat of reprimand following findings from clinical audits. Other research has shared similar concerns that the fear of blame/reprimand, the shortage of resources and expertise to implement changes and the lack of manpower for effective supervision amongst others are capable of adversely affecting the morale to conduct clinical audits [19,22]. Additionally, diminished clinical ownership, restriction of clinical freedom and fear of litigation were some disadvantages of clinical audit reported by Johnston *et al*. [23]. The finding implies that this would continue to instigate the decay being experienced in the health care system until a bold move can be initiated by concerned stakeholders to ensure that a conducive atmosphere exists for routine conduct of clinical audits as essential interventions for enhanced service delivery. It is also worthy of note that the conduct of multidisciplinary audit is more successfully established in areas that are already predisposed towards team-working or where paramedical health professionals have high involvement in decision-making [19]. In this study, only a small percentage of physicians participated in clinical audits. This low participation could be reflective of the dearth of training and poor emphasis placed on CA activities. The push factor in places where higher participation in clinical audit activities has been reported, like in the United Kingdom and Australia had been the institutional requirements and support for clinical audit [20,21].

This study provided useful data on the level of implementation of clinical audit in a developing health system. Clear imperatives from the findings of this study are the need for improvement in the knowledge, attitude and the level of involvement of doctors and other members of the health team. In tertiary health facilities in this setting embroiled in persisting intra-professional wrangling [14], the institutionalization of multidisciplinary teams for clinical audit could become a veritable tool for fostering teamwork and the achievement of improved results in the health care system [22]. Future research should focus on the development of local standards and strategies for institutionalizing clinical audit in health service organizations. Innovative approaches to incentivizing involvement in clinical audits by health care providers should also be explored.

**Limitation of the study:** convenience sampling method adopted in this study may have contributed to some bias. Therefore, findings from this study may not be generalizable to all doctors in the Teaching hospital.

## Conclusion

Findings from this study revealed that while most physicians had a positive attitude toward the various aspects of clinical audit, their knowledge of the clinical audit process was poor and only a small percentage had been involved with clinical audits. The institutionalization of clinical audit in local health care practice and periodic training of physicians on clinical audit is recommended.

### What is known about this topic

- Clinical audit involves the process of comparing current practice to evidence-based best practice in the form of standards of care, identifying areas for quality improvement and implementing changes to meet the standards;
- As a quality improvement tool, clinical audit demonstrates accountability and shows that effort is being made by dedicated health professionals to deliver high-quality care to patients.

### What this study adds

- This study highlights the need for the initialization of a systematic program of routine clinical audit into health care practice. The initiation of routine clinical audit at different levels of clinical governance within the health care system of Nigeria irrespective of the various concerns that could hinder the implementation of recommended action plans cannot be over-emphasized;
- Early adoption of audit-oriented medical practice may be achieved by including clinical audit as a course in the medical school curriculum.
